# Achromatic Markings as Male Quality Indicators in a Crepuscular Bird

**DOI:** 10.3390/biology14030298

**Published:** 2025-03-16

**Authors:** Richard Schnürmacher, Rhune Vanden Eynde, Jitse Creemers, Eddy Ulenaers, Marcel Eens, Ruben Evens, Michiel Lathouwers

**Affiliations:** 1 Behavioural Ecology and Ecophysiology Research Group, Department of Biology, University of Antwerp, Universiteitsplein 1, 2610 Wilrijk, Belgium; jitse.creemers@uantwerpen.be (J.C.); marcel.eens@uantwerpen.be (M.E.); ruben.evens@uclouvain.be (R.E.); 2 Department of Zoology, Faculty of Natural Sciences, Comenius University, Ilkovičova 6, 842 15 Bratislava, Slovakia; 3 Research Group: Zoology, Biodiversity and Toxicology, Centre for Environmental Sciences, Hasselt University, Campus Diepenbeek, Agoralaan, Gebouw D, 3590 Diepenbeek, Belgium; rhune.vandeneynde@student.uhasselt.be (R.V.E.); michiel.lathouwers@uhasselt.be (M.L.); 4 Terrestrial Ecology and Biodiversity Conservation Group, Earth and Life Institute, Université Catholique de Louvain, Croix du Sud 4-5, 1348 Louvain-la-Neuve, Belgium; 5 Agentschap Natuur en Bos, Regio Noord-Limburg, Heuvelstraat 1C, 3941 Hechel-Eksel, Belgium; eddy.ulenaers@vlaanderen.be; 6 Department of Geography, Institute of Life, Earth and Environment (ILEE), University of Namur, 61 Rue de Bruxelles, 5000 Namur, Belgium

**Keywords:** honest signalling, sexual selection, European Nightjar, ornaments, age-related changes, body condition, site fidelity

## Abstract

Secondary sexual traits, such as specific body parts or colouration, play an important role in mating interactions. It has been proposed that they function as quality indicators driven by sexual selection. In birds, much attention has been paid to the study of feather pigmentation, especially in diurnal passerines. However, recent research demonstrates that structural achromatic colours are likely to be of similar importance for communication, especially for species inhabiting poorly lit environments and that are active at night. Using 15 years of capture–recapture data from a long-term study on adult European Nightjars (*Caprimulgus europaeus*), we investigated the role of males’ white tail and wing markings as secondary sexual traits. We show that the inter-individual variation in marking size exceeds that of the other morphometric variables, suggesting that wing and tail markings could be subject to sexual selection. Older males, individuals with a higher body condition index, and long-term territory holders had larger markings, while these effects were particularly pronounced in terminal tail feather markings. The importance of markings for signalling is likely related to their observed use in social displays. Pronounced site differences in tail marking sizes and annual variation suggest environmental factors acting on the ornaments that remain to be further examined.

## Simple Summary

Many animals use conspicuous body parts in communication, often as indicators of individual quality. While this has been extensively studied in the colourful plumage of diurnal songbirds, little is known about the role of contrasting white markings in nocturnal species. Over 15 years, we captured European Nightjars, nocturnal birds with distinct white markings on their wings and tail in males. We measured these markings and examined whether they show greater variability than other body parts, suggesting sexual selection pressures on their size. We also investigated the associations between the size of these markings and indicators of individual quality, such as age, body condition, site fidelity, and whether these patterns varied across study sites and between years. Our findings revealed that larger markings, particularly in the tail feathers, were associated with older males, those in better condition, and males returning to the same breeding site. The marking size varied across study sites. Given the visibility of these markings during breeding and territorial displays of Nightjars, we propose that they act as quality indicators. This study highlights the potential role of white markings in the communication of birds active at night, advancing our understanding of sexual selection in these enigmatic species.

## 1. Introduction

Secondary sexual traits allow honest intra- and intersexual communication about individual quality signalled through these traits [1]. Unlike primary sexual traits, such as gonads, secondary sexual traits, including specific structures or conspicuous colours, do not directly facilitate reproduction. However, they inform potential rivals and partners about an individual’s physical ability to reproduce and its phenotypic quality. This link between secondary sexual traits, individual quality and mate preference has driven research in sexual selection since the development of this concept [1,2,3,4].

Sexual selection promotes the improvement of clarity, visibility and comprehensibility of the honest signals used for mate attraction [5,6]. A signal is deemed ‘honest’ if it reliably conveys information about its bearer, either because it is costly to produce or is inherently tied to physiological processes that cannot be counterfeited [7]. This allows the ‘choosy’ sex, represented by females in most avian taxa, to assess potential mates according to the quality of their secondary sexual traits. In birds, these traits, either acoustic or visual, are often produced simultaneously in complex displays. Among visual cues, feather morphology (i.e., length, width, structure and shape of feathers) and colouration, collectively termed ornamentation, show an exceptional diversity. Many diurnal birds use bright colours to attract potential mates [8,9]. In species inhabiting dark environments, such as dense forests [5,10], or in animals with crepuscular and nocturnal lifestyles [10,11], pigment-free white markings on a dark background have evolved to serve this role. Similarly to diurnal species, such as Collared Flycatchers (*Ficedula albicollis*), Pied Flycatchers (*Ficedula hypoleuca*) and Barn Swallows (*Hirundo rustica*) [12,13,14,15], contrasting patterns of black-and-white maximise the light reflectance and detectability in dim light conditions.

Research on the function of secondary sexual traits has focused primarily on their role in diurnal species, whereas their functioning in crepuscular and nocturnal species remains largely overlooked [11]. In the past decade, however, several studies have highlighted the signalling importance of visual cues in nocturnal non-passerines; including owls [16], storm petrels [17] and waders [18]. For instance, in male Eagle Owls (*Bubo bubo*), the reflectance of a large white throat patch peaks during the breeding season [19], potentially aiding in the assessment of intruder and/or mate quality [16,20]. Moreover, males also predominantly display these white throat patches around twilight and during moonlit nights [21].

Caprimulgidae, comprising approximately 98 crepuscular and/or nocturnal aerial insectivores [22], exhibit cryptic colourations for camouflage [23]. Within this family, males, and occasionally both sexes, possess white markings on their wings and tail, accompanied by white throat patches in some species. These markings likely serve roles in communication during mating and territorial displays [24]. The evolution of conspicuous achromatic (pigment-free) markings in Nightjars may reflect a trade-off between effective signalling and predator avoidance [25,26]. As visually oriented birds, Nightjars rely on adequate ambient light levels to be active, which is likely linked to the assessment of these visual signals [27,28,29]. To date, several studies have proposed that caprimulgids use these markings as indicators of individual quality. Still, the knowledge on factors influencing variation in the white markings of caprimulgids is very limited. Although several studies have described intra- and intersexual differences between juveniles and sexually mature individuals [26,30,31,32,33], only one [34] has demonstrated that the size of the ornaments of wing and tail bands in Common Nighthawks (*Chordeiles minor*) increases between yearlings (birds in their second calendar year; 2CY) and males older than the second calendar year (>2CY), with no additional factors examined.

**Figure 1:**
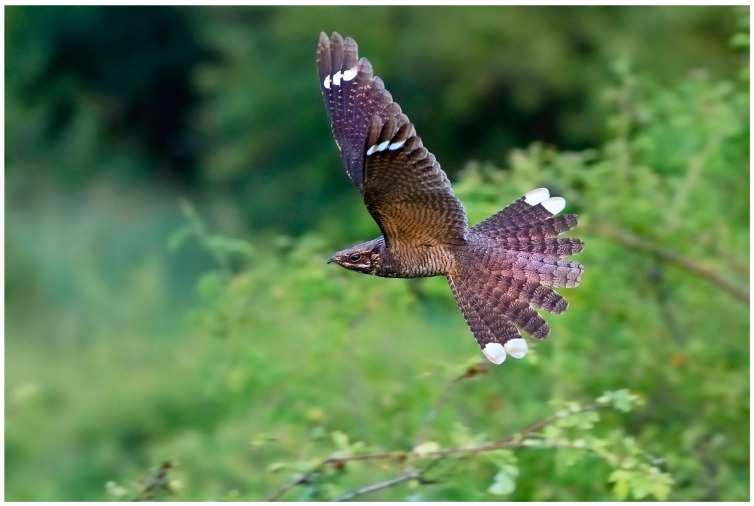
Male European Nightjar during a territorial display, showing its white markings (photo credit: Ervín Hrtan, with permission).

The European Nightjar (*Caprimulgus europaeus*; hereafter Nightjar) inhabits an extensive breeding range across Eurasia [35] and overwinters in sub-Saharan Africa [36,37]. Nightjars are sexually dichromatic with adult males possessing subterminal white spots on the three outer primaries and terminal white tips on the two outermost tail feathers (hereafter ‘rectrices’). These markings are less pronounced and never pure white in females. In less than 1% of males, an additional white marking can develop on P7 or T3 (personal observations; Supplementary Materials: Figure S1). The white markings are visible mainly in flight and are prominently displayed during courtship and territorial defence, when males glide with V-shaped wings and fan their tail feathers towards the rival or potential partner ([38,39]; see also Figure 1).

To understand the potential role of achromatic ornaments in nocturnal birds, we investigate whether male markings serve as sexually selected traits by testing the hypothesis that their size variability exceeds that of other morphometric traits [40,41]. We also examine whether these markings are honest indicators of male quality by studying associations between marking size and individual age, site fidelity and body condition, as well as considering site-specific differences. To achieve this, we used a long-term dataset spanning 15 years of feather markings of male Nightjars in Belgium.

## 2. Materials and Methods

### 2.1. Data Collection

During the breeding season (2010–2024; April–September) we captured male Nightjars in four main study populations in Belgium: National Park Bosland (‘Bosland’; 51°11′ N 5°20′ E), Grenspark Kalmthoutse Heide (‘Kalmthout’; 51°23′ N 4°25′ E), National Park Hoge Kempen (‘NPHK’; 50°58′ N, 5°37′ E) and Oudsbergen Military Area (‘MDME’; 51°2′ N, 5°27′ E). The individuals were captured using ultra-fine mist nets (15 × 3 m) and tape lures [42]. We marked each captured bird with a unique alphanumeric ring. Ringing activities were performed by licensed ringers following standard procedures of the Royal Belgian Institute of Natural Sciences and complied with ethical standards for capturing birds.

For this study, we classified adult males based on their relative age at the time of ringing, as follows: yearlings (2CY), or older males (>2CY). When it was impossible to distinguish between 2CY and >2CY, the relative age at ringing date was recorded as older than the first calendar year (>1CY). This situation arises when individuals have completed a full winter moult and no distinction could be made between retained and moulted feathers (alula, coverts, secondaries), hampering more precise age determination [43]. In further analyses, these age classes were treated separately (Supplementary Materials: Tables S1 and S2). If an individual was captured in more than one year, we also calculated its minimum known age based on the relative age at the ringing date. For instance, an individual aged as >2CY (at least 3 years of age) in 2012 would be at least 5 years old if recaptured in 2014. Additionally, we assessed an individual’s site fidelity as a binary variable (yes/no), based on whether it was recaptured at the same breeding site in the following years. All records of birds recaptured at the same site at least once were considered ‘returners’ (including initial capture).

We measured the size of the white markings in three outermost primaries (P8–P10) and two outermost rectrices (T4, T5; see also Supplementary Materials: Figure S1). Two methods—manual (M) and digital (D)—were applied to determine the size of these markings. From 2010 to 2018, we manually measured the width and height of the inner and outer web of the P8 markings (Supplementary Materials: Figure S2a) and the height of the markings in T4 and T5 using a digital calliper (Supplementary Materials: Figure S2b). We estimated the surface area of the P8 markings by multiplying the measured width and height [34]. Since 2019, we have digitally measured the size of all the wing (P8–P10) and tail markings (T4, T5) from photographs using ImageJversion 1.54 (https://imagej.net/ij/ (accessed on 3 December 2023); Supplementary Materials: Figure S3). Following a standardised procedure, we recorded the total surface area of the selected marking in mm^2^.

Throughout the study, wing length (chord) and body weight were measured for all individuals. The tail length (T5 and T1) and sternum (keel) length as a skeletal predictor of body size [44] were noted only for individuals whose markings were measured manually (2010–2018). We estimated the body condition using the ordinary least squares (OLS) method as the residuals of a regression of the wing length on the body mass for each individual [45,46,47], following previous studies on caprimulgids [48,49].

To evaluate the comparability of the manual and digital measurements and left–right symmetry, we quantified the wing and tail markings of 22 male Nightjars in Royal Belgian Institute of Natural Sciences (RBINS, Brussels) collections using both methods [50].

### 2.2. Statistical Analyses

#### 2.2.1. Analyses of Museum Specimens

We correlated manually and digitally measured values of individual markings using linear regression. Additionally, we tested the right–left wing and tail symmetry of each marking using a paired *t*-test. Because of the high degree of intra-individual symmetry (*p*-value ranging from 0.95 to 0.45; Supplementary Materials: Table S3), we decided to use only the wing and tail markings on the left side of the body for the field-collected data.

#### 2.2.2. Markings as Sexually Selected Traits

To investigate whether male markings may be subject to sexual selection, we compared the inter-individual variation in markings with other morphometric traits. We calculated the coefficient of variation (ratio of the standard and mean multiplied by 100) for each morphometric trait (weight, sternum length, length of T5 and T1, wing length) and for each marking (T4M, T5M and P8M; T4D, T5D, P8D, P9D and P10D) using base R [51]. To test for significant differences between these coefficients, we used the modified signed-likelihood ratio test (MSLRT) of the R package cvequality, version 0.2.0 [52,53].

#### 2.2.3. Markings as Honest Quality Indicators

To investigate the honesty of markings as quality indicators, we constructed a generalised linear mixed model (GLMM) for each combination of feather marking (T4, T5; P8, P9 and P10) with a measurement type (manual or digital). We constructed separate models due to collinearity reasons and differences in used metrics for the respective feathers (mm vs. mm^2^). Each model explored whether a feather marking was influenced by a main predictor, as follows: relative age (categorical variable: 2CY, >1CY, >2CY), minimum known age (categorical variable: 2 to >5 years), site fidelity (return to the same territory; binary variable: yes/no) or body condition (continuous variables). In each model, we controlled for the study site (categorical variable: Bosland, Kalmthout, MDME, NPHK) and included individual identity and year as random intercepts. Since the study site significantly influenced the size of the tail markings as a covariate, we decided to also include site as a main predictor in the final set of models. Using backward elimination, we compared likelihood ratio tests (LRT) and AICs of full models and models with removed factors or interactions, excluding insignificant factors (*p* > 0.05), and selecting final models [54].

All models were performed using functions from the glmmTMB package, version 1.1.8 [55]. Univariate GLMMs and Type III analyses of variance (ANOVA) quantified relationships between the marking size and explanatory variables. For categorical predictors with more than two levels, we performed Sidak post hoc tests adjusted for multiple comparisons of the emmeans package, version 1.9.0 [56]. The model specification problems were assessed using the package DHARMa, version 0.4.6 [57].

Because the ornament size of T4D and T5D differed significantly between two main study sites (see Results), to explore the within-season variation on a continuous scale (Julian Day), we tested the changes in marking size using generalised additive mixed models (GAMMs) to evaluate whether the local differences are constant across the breeding season or follow a non-linear pattern during certain periods of the season. We included individual and year as random effects to account for individual and annual variability.

## 3. Results

In total, we measured the size of wing and tail markings of 736 males, including 166 individuals captured in more than one year (see Supplementary Materials: Tables S1 and S2). We measured the markings of 265 individuals manually and 490 individuals digitally (Table 1). For 19 individuals, the marking size was measured using both methods, although not in the same year.

Based on 22 museum specimens, we determined that the corresponding marking sizes on both sides of the body are nearly identical within individuals and that there were significant and mostly strong associations between manual and digital measurements, although the strength of the correlations differed among feather types (ranging from P8: adjusted R^2^ = 0.878 to T5: adjusted R^2^ = 0.291; see Supplementary Materials: Table S3). However, we used these results only to justify using one-side measurements of each individual and we do not elaborate on symmetry further in this study.

### 3.1. Markings as Sexually Selected Traits

**Figure 2:**
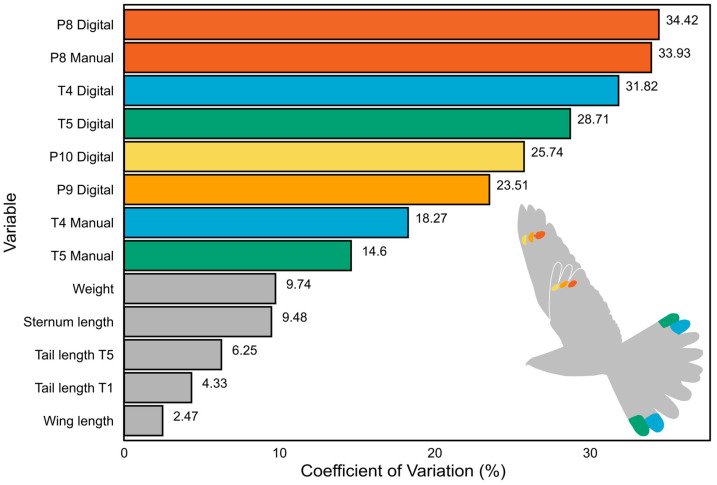
Comparison of the coefficient of variation (CV) values for marking sizes and other morphometric traits. The values at the end of each bar represent the CV of the corresponding variable. Note that all marking sizes exhibit higher CV values than other traits, indicating a greater likelihood that sexual selection pressure acts on the markings. The colour of the bars corresponds to the respective feather markings (T4—blue, T5—green, P8—dark orange, P9—light orange, P10—yellow); the non-ornamental body traits are grey-coloured.

The coefficients of variation differed significantly in all markings (14.60% to 34.42%) compared to morphological traits (2.47% to 9.74%; see also Figure 2; Supplementary Materials: Table S4). The inter-individual variation in marking size was on average four times higher (26.42% vs. 6.45%) than the variation in other morphological traits, with the highest variance in the size of P8M and P8D and the surface area of T4 and T5 (Figure 2).

### 3.2. Markings as Honest Quality Indicators

**Figure 3:**
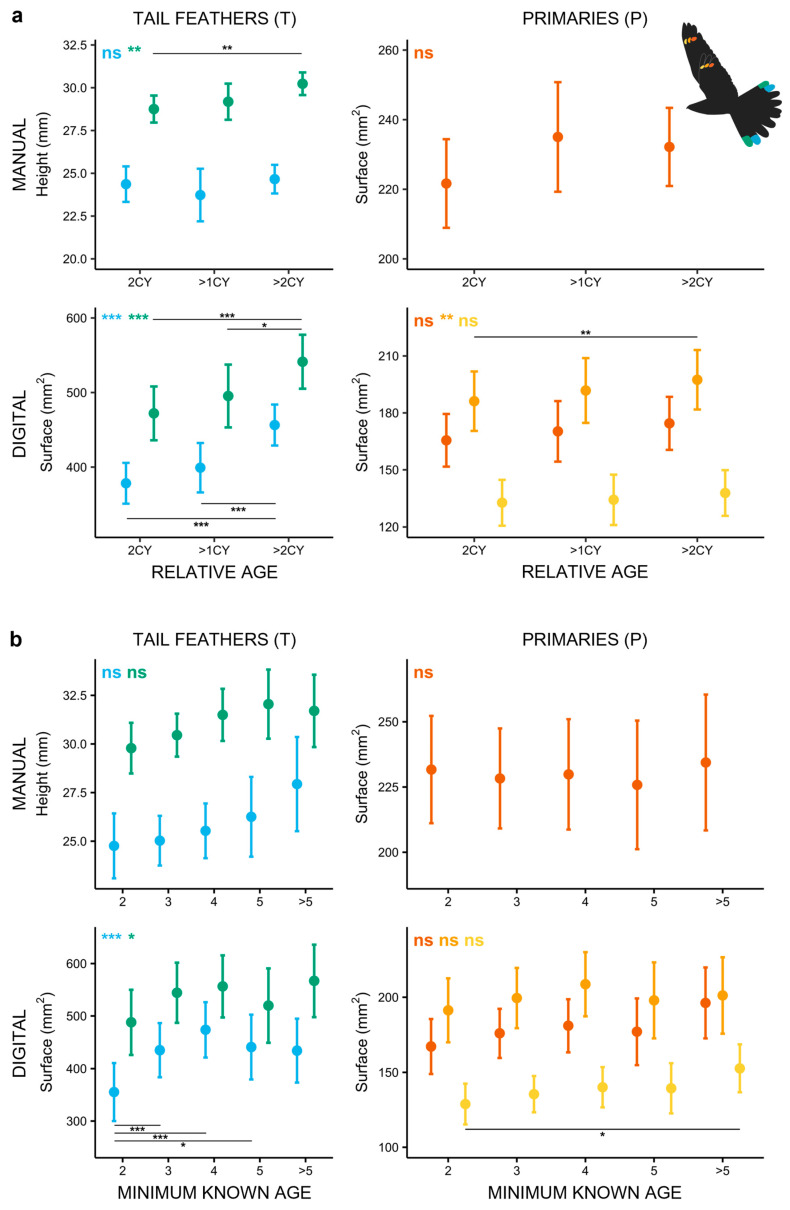
Marking size of male Nightjars in relation to: (**a**) relative age (full dataset) and (**b**) minimum known age (based on recapture data). Older males generally have larger markings, especially in the rectrices. Notable increases in the marking size with minimum known age are evident in the surface area of the tail markings. Pairwise tests reveal a significant increase in T4D ornament size between 2CY and older birds. Inscriptions in the upper left corner indicate the overall significance of the models with matching colours, horizontal lines with asterisks mark significant post hoc results. (“*” corresponds to *p* < 0.05; “**” corresponds to *p* < 0.01; “***” corresponds to *p* < 0.001); “ns” corresponds to a non-significant result (*p* > 0.05). The colours correspond to the respective feather markings in the flying Nightjar icon.

Our long-term data demonstrate that older males (>2CY) have larger markings than yearling males (2CY) and than individuals whose relative age could not be determined (>1CY; Figure 3a; Supplementary Materials: Tables S5 and S6). This effect was most pronounced in tail markings (T5M: *χ*^2^ = 10.030, *p* = 0.007; T4D: *χ*^2^ = 54.920, *p* < 0.001; T5D: *χ*^2^ = 32.589, *p* < 0.001) and in one primary (P9D; *χ*^2^ = 9.731, *p* = 0.008).

Building further on these differences in tail markings between yearling and older individuals, but not between 2CY and >1CY birds (Figure 3a; Supplementary Materials: Figure S4a), we attempted to understand whether the >1CY group in fact comprised yearling individuals. This hypothesis was supported by data from recaptured males with a known minimum age. Of the recaptured birds aged as >1CY (*N* = 44), only 13 individuals were already ringed initially as adults, while most (31 of 44; 70.45%) were ringed in the previous year as juveniles (1CY; Supplementary Materials: Figure S4b). This cohort of 31 birds recaptured as >1CY represented nearly 40% of the actual 2CY birds ringed as juveniles (81 individuals; Supplementary Materials: Figure S4c).

When considering the influence of minimum known age, the marking size suggests that tail markings grow until males reach 4 years of age, with a non-significant trend in wing markings (Figure 3b). Models containing the presumed minimum age of individuals suggest that the size of the tail markings increases with age (T5M: *χ*^2^ = 9.300, *p* = 0.054; T4D: *χ*^2^ = 29.743, *p* < 0.001; T5D: *χ*^2^ = 9.956, *p* = 0.041; Supplementary Materials: Tables S7 and S8). However, the post hoc tests indicate significant size differences only between markings of yearling birds (2CY) and older cohorts. In accordance with this, a comparison of intra-individual changes in ornament sizes in a subset of birds captured as yearlings and then in subsequent years as older males show marked increases in the length of T5M (*χ*^2^ = 13.416, *p* < 0.001) and the surface area of T4D (*χ*^2^ = 12.078, *p* < 0.001).

**Figure 4:**
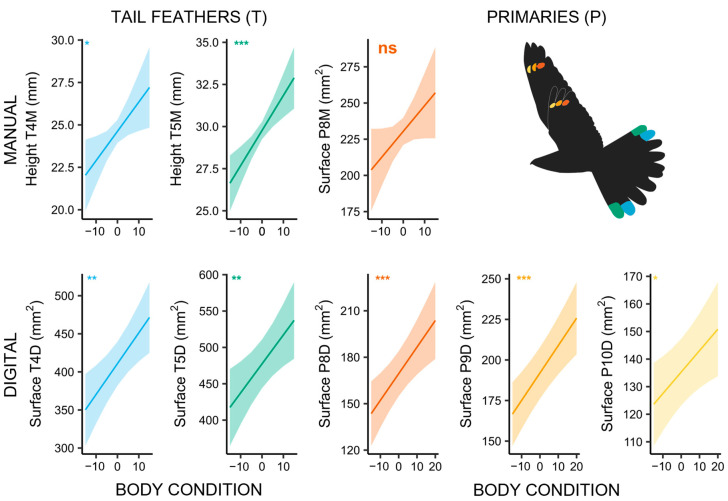
Marking size of male Nightjars in relation to their body condition index, showing a strong correlation between larger markings and higher body condition. Inscriptions in the upper left corner indicate the overall significance of the models with matching colours (“*” corresponds to *p* < 0.05; “**” corresponds to *p* < 0.01; “***” corresponds to *p* < 0.001); “ns” corresponds to a non-significant result (*p* > 0.05). The colours correspond to the respective feather markings in the flying Nightjar icon.

Our results further show that males with a higher body condition have significantly larger markings (T4M: *χ*^2^ = 5.658, *p* = 0.017; T5M: *χ*^2^ = 13.617, *p* < 0.001; T4D: *χ*^2^ = 10.367, *p* = 0.001; T5D: *χ*^2^ = 8.241, *p* = 0.004; P8D: *χ*^2^ = 11.285, *p* < 0.001; P9D: *χ*^2^ = 18.172, *p* < 0.001; P10D *χ*^2^ = 6.417, *p* = 0.011; Figure 4; Supplementary Materials: Tables S9 and S10). Individuals that were recaptured within their territory in the following years also had larger tail markings than individuals that did not return to the same area or that did not return at all (T4M: *χ*^2^ = 6.953, *p* = 0.008; T5M: *χ*^2^ = 10.664, *p* = 0.001; Supplementary Materials: Figure S5; Tables S11 and S12).

Finally, we observed that the tail marking size differed between study sites (T4D: *χ*^2^ = 14.323, *p* = 0.002; T5D: *χ*^2^ = 8.981, *p* = 0.030; Supplementary Materials: Figure S6; Tables S13 and S14), with males from Bosland having significantly larger markings than MDME males (T4D: Estimate = −47.931, *p* < 0.001; T5D: Estimate = −57.839, *p* < 0.001; Supplementary Materials: Figure S7). An additional analysis, investigating the population-level trend in marking size in both study sites, suggests that throughout the season, patch size was relatively constant in Bosland (T4D: edf = 1.719, *F* = 1.716, *p* = 0.111; T5D: edf = 2.646, *F* = 0.446, *p* = 0.691), whereas this trend was not the same in MDME (T4D: edf = 3.827, *F* = 3.315, *p* = 0.022; T5D: edf = 2.693, *F* = 3.627, *p* = 0.056). In MDME, we observed notable reductions in population-level patch size in early June and in August, especially in T4D (Supplementary Materials: Figure S7).

## 4. Discussion

Our analysis of white markings in the primaries and rectrices of 736 adult male Nightjars indicates that specific markings can be considered ornaments under sexual selection. In particular, the size of tail ornament T4 was larger in older males, in males with a higher body condition and in males that were captured and recaptured in following years at the same site. Our data also suggest that ornament size is influenced by environmental factors, but these have yet to be identified.

### 4.1. Markings as Sexually Selected Traits

In our study, the coefficient of variation in feather markings was significantly higher than that observed in morphometric traits, such as wing and tail length (Supplementary Materials: Table S4). In sexually dimorphic species, such as Nightjars, this suggests that white feather markings are sexually selected secondary traits and can be considered ornaments [40,58]. Moreover, the size of the innermost markings (T4 and P8) showed the highest variation among ornaments (Figure 2), suggesting their importance during social interactions [40,58].

The discrepancy in variability between ornamentation and other morphometric characteristics is generally attributed to the influence of directional selection on ornaments and their condition-dependent expression, reflecting female preference and stabilising selection on non-sexual characteristics [59,60,61]. However, the high variation in an achromatic marking does not universally indicate that a trait is strongly influenced by sexual selection. As demonstrated in the ornaments of male Pied Flycatchers, where the white forehead patch and dark plumage are considered ornaments, the strength of sexual selection pressure can vary across populations due to climate conditions and differences in male–male competition, especially in the edge of the species range [62,63,64].

From a behavioural perspective, the detectability (size, shape and brightness) of contrasting ornaments in low-light environments is essential for precise communication and quality assessment [11]. Nightjars’ mating and territorial behaviour suggests that white markings in males’ wings and tail convey information for rivals and potential mates. During their display, male Nightjars glide over their territory, with fanned wings and tail, exhibiting their white markings [39,65] (see also Figure 1). When male Nightjars are not displaying, the innermost markings are partially covered by outer feathers, which may, in addition to concealing the visibility of conspicuous markings to predators [23,25], aid in resisting degradation better compared to the rest of the markings [15,66,67].

### 4.2. Markings as Honest Quality Indicators

#### 4.2.1. Age

We observed that the size of the tail ornaments increases with relative age, with the most pronounced size differences between yearlings (2CY) and older (>2CY) males. Further investigation of individuals that completed a full winter moult (>1CY) suggested that most of these birds are just 2CY males with marginally larger tail ornaments than other yearling males, but their ornaments are still significantly smaller than those of older males. The >1CY individuals underwent a complete moult in Africa, although the reason for why Nightjars moult completely or retain certain feathers is unknown [68]. However, based on recapture data, almost 30% of the recaptured birds aged in the field as >1CY were actually older individuals. Therefore, it is not possible to conclude that all Nightjars which moult their feathers completely are yearling birds.

In the case of yearling males that were later also recaptured as older males, ornaments of T4D were significantly smaller in their first summer than in subsequent years. Moreover, for all the other individuals whose minimum age could be estimated, our data also suggest that the size of tail ornaments (especially T4) peaks when individuals are at least 4 years old and then decreases slightly in older individuals (≥5 years; Figure 3b). This may correspond to the findings that Nightjars are relatively short-lived birds with an average lifespan of 4 years, although some individuals may reach 8–10 years of age ([69]; personal observations). Overall, smaller ornament size may therefore possibly indicate inexperienced yearling males [34,70,71,72] and some form of senescence in very old individuals [73,74].

#### 4.2.2. Body Condition and Site Fidelity

Our data demonstrate that males with a higher body condition index have larger ornaments. This supports the hypothesis that white ornaments can serve as honest signals that convey individual quality [3,15,75]. As shown in other species, the ornament size may indicate territory quality, aggressiveness and social status of an individual [76,77]. In general, older, more experienced males and successful breeders have a higher probability of returning to the breeding site [78,79,80]. This may explain why, in our study, individuals that returned to the same breeding site in the following years had larger tail ornaments than individuals that did not return [3,80,81].

However, it should be noted that the size of male ornaments reflects the individual body condition during feather growth, which in the case of Nightjars occurs during the wintering season [82]. This may suggest that benign winter conditions could lead to a higher condition from which individuals benefit in later stages of the annual cycle. To investigate this would require population-level data on wintering conditions (see [14,83]), but that falls beyond the scope of this study.

#### 4.2.3. The Influence of Environmental Parameters on Marking Size

The ornament size fluctuated between years, suggesting that several environmental parameters, such as weather and prey availability or differences in age-group related annual survival rate, may contribute to the additional population-level variation. At this moment, it is unclear which parameters it concerns; however, it further supports the idea that ornament development is not solely influenced by genetic factors, but also by how the individual is influenced by the environment during feather growth [14,84,85]. Therefore, further research on these environmental variables, especially at wintering grounds, is necessary to evaluate their impact.

We also discovered that tail ornaments are significantly smaller in MDME compared to National Park Bosland (Supplementary Materials: Figure S6; Tables S13 and S14), two key study sites 15 km apart, accounting for 33.1% and 44.1% of the observations, respectively. This size disparity was not explained by differences in general age class proportions between sites (*t*-test comparisons non-significant; 2CY: *t* = −0.399, *p* = 0.698; >1CY: *t* = 1.022, *p* = 0.331; >2CY: *t* = −0.382, *p* = 0.712), though slightly more >1CY males were captured in Bosland (Bosland vs. MDME: 2CY = 36.13 vs. 39.56%, >1CY = 17.02 vs. 12.66%, >2CY = 46.85 vs. 47.78%; 429 vs. 316 captures).

We can speculate that habitat differences between the sites may create different selection pressures on ornament sizes. Bosland, for example, predominantly comprises small forest clearings in managed pine plantations, while territories in MDME are located in open heathlands within a military area. In darker, denser habitats like Bosland, selection may favour more conspicuous ornaments for signal visibility, potentially explaining larger markings [8,11]. However, we have no direct evidence for an effect of light environment on ornament development, apart from the observed larger size in birds originating from the site with more enclosed vegetation (Bosland). Other environmental factors, such as diet and exposure to stressors [63,84], may play an equally important role in determining the ornament size. Therefore, this aspect warrants further investigation.

Other than the smaller ornaments in MDME compared to National Park Bosland, our data also demonstrate two seasonal decreases in ornament size of captured individuals in MDME (Julian Day 213–243; Supplementary Materials: Figure S7). These periods coincide with increased captures of 2CY males (Supplementary Materials: Figure S8), suggesting an influx of yearlings and/or floaters (May–June) or single-brood breeders (July–September; [86]), which deserves further study.

### 4.3. Limitations and Future Perspectives

Manual and digital measurements of museum specimens have shown a high correlation in determining the height and surface area of the ornaments of the respective feathers. However, in the case of T5, the correlation was considerably lower. We argue that this could have been caused by extensive abrasion of the outer tail feathers, which is less apparent in the inner feathers (T4), along with different widths of the T5. The digital method encompasses higher variability, which accounts for these differences in feather width. Alternatively, the length of the ornament may be used, provided that the tip of the feather rachis is intact. Therefore, to obtain comparable results, we advise the use of standardised conditions for photographing the feathers (ideally fully spread) and omitting individuals with highly damaged feather vanes from the analyses, as we did with field data [50].

In recaptured birds initially ringed as >1CY or >2CY, we were able to determine only their minimum known age, while the actual age of these individuals could have been higher [87]. In future studies, additional factors related to body condition (including skeletal metrics, haematocrit and parasite load) and reproductive success would further refine quality assessments [66]. Other selection pressures not tested in this study may also limit the size of the ornaments, such as conspicuousness to predators [26] and structural weakness of achromatic plumage [66,67]. Finally, gathering more detailed environmental data from wintering grounds could clarify how the environment influences marking development [73,85].

## 5. Conclusions

Our long-term dataset on male white wing and tail markings suggests that Nightjars’ achromatic markings can be considered sexually selected traits. The variation observed in the white markings significantly exceeds the variation in other body measurements, suggesting ongoing sexual selection and their role as ornaments. The height and surface area of tail ornaments significantly increases with age, body condition and is largest in males that return to a territory in subsequent years, with a weaker effect observed for ornaments on primaries. Ornament size also varied between breeding sites, hinting towards a higher prevalence of males with larger tail ornaments at the darker sites. We conclude that ornaments of male European Nightjars likely act as honest quality indicators, conveying information on their individual status to potential female partners and male rivals. Nonetheless, much of the variation remains to be explained, highlighting future challenges to understand visual signalling in caprimulgids, as well as in other species active at twilight and night.

## Figures and Tables

**Table 1:** Distribution of height (T4M, T5M) and surface area (P8M; T4D, T5D, P8D, P9D, P10D) of white markings in male Nightjars (*N* = 955 between-year captures of 736 unique birds). ‘M’ stands for manual and ‘D’ for digital measurements (*N_M_* = 343 captures; *N_D_* = 612 captures).

Feather	Manual (mm)			Digital (mm^2^)		
	Min.	$\bar{x}$	Max.	Min.	$\bar{x}$	Max.
T4	8.88	24.55	36.97	16.12	387.85	777.87
T5	8.72	29.85	43.61	102.6	486.00	926.40
P8 ^a^	21.85	232.35	438.46	23.40	173.30	466.42
P9	-	-	-	44.25	197.36	382.34
P10	-	-	-	27.43	139.36	250.87

^a^ The value for P8M is expressed in mm^2^.

## Data Availability

The raw data supporting the conclusions of this article will be made available by the authors on request.

## Supplementary Materials

The following supporting information can be downloaded at: https://www.mdpi.com/article/10.3390/biology14030298/s1, Figure S1: White markings on the outer (a) primaries (P8–P10) and (b) rectrices (T4, T5) of a male Nightjar; Figure S2: Manual measurements of feather markings; Figure S3: The protocol followed to obtain digital measurements of the feather markings; Figure S4: (a) Density plots of the tail feather surface of male Nightjars by age group, suggesting that >1CY individuals comprise both 2CY and >2CY birds, with more individuals originating from 2CY cohort. This is supported by the categorisation of these individuals in data on recaptured individuals. (b) Most recaptured individuals aged as >1CY were, in fact, 2CYs based on their age during the initial ringing date. (c) On the contrary, >1CY birds form a significant proportion of actual yearling birds from the recapture data; Figure S5: Marking size of male Nightjars in relation to their site fidelity; Figure S6: Marking size differences of male Nightjars between study sites; Figure S7: Comparison of changes in within-year marking size between Bosland and MDME by date of capture, showing greater fluctuation in MDME; Figure S8: Ratios of >2CY (positive values) and 2CY (negative values) of male Nightjars caught throughout the season in Bosland (red circles) and MDME (blue circles); Table S1. Overview of the total number of male European Nightjars captured each year at the respective study sites by age group; Table S2. Overview of the total number of male European Nightjars recaptured across years at the respective study sites by age group; Table S3. Results of compared values obtained from museum male Nightjar specimens (N = 22); Table S4. Pairwise comparisons of modified signed-likelihood ratio tests (MSLRTs) for coefficient of variation; Table S5. Results of generalised linear mixed models, type III analyses of variance and significant post hoc tests, showing effects of relative age on size of the white tail markings of male Nightjars; Table S6. Results of generalised linear mixed models, type III analyses of variance and significant post hoc tests, showing effects of relative age on size of the white wing markings of male Nightjars; Table S7. Results of generalised linear mixed models, type III analyses of variance and significant post hoc tests, showing effects of minimum known age on size of the white tail markings of male Nightjars; Table S8. Results of generalised linear mixed models, type III analyses of variance and significant post hoc tests, showing effects of minimum known age on size of the white tail markings of male Nightjars; Table S9. Results of generalised linear mixed models, type III analyses of variance and significant post hoc tests, showing effects of body condition index on size of the white tail markings of male Nightjars; Table S10. Results of generalised linear mixed models and type III analyses of variance, showing effects of body condition index on size of the white wing markings of male Nightjars; Table S11. Results of generalised linear mixed models, type III analyses of variance and significant post hoc tests, showing effects of site fidelity (recapture: yes/no) on size of the white tail markings of male Nightjars; Table S12. Results of generalised linear mixed models and type III analyses of variance, showing effects of site fidelity (recapture: yes/no) on size of the white wing markings of male Nightjars; Table S13. Results of generalised linear mixed models, type III analyses of variance and significant post hoc tests, showing effects of the study site on size of the white tail markings of male Nightjars; Table S14. Results of generalised linear mixed models and type III analyses of variance, showing the effects of the study site on size of the white wing markings of male Nightjars.
