# Supplementary material for: Achromatic Markings as Male Quality Indicators in a Crepuscular Bird
Source: Biology (Basel). 2025 Mar 16;14(3):298. doi: 10.3390/biology14030298 (PMC11940135; doi:10.3390/biology14030298)
Supplement: Supplementary file 1 [file biology-14-00298-s001.zip › biology-3513852-supplementary.pdf]

Achromatic tail markings as male quality indicators in a crepuscular bird

Richard Schnürmacher [1,2†*], Rhune Vanden Eynde [3†], Jitse Creemers [1,4], Eddy Ulenaers [5], Marcel Eens [1], Ruben Evens [1,4‡], Michiel Lathouwers [3,6‡]

1 Behavioural Ecology and Ecophysiology Research Group, Department of Biology, University of Antwerp, Universiteitsplein 1, 2610 Wilrijk, Belgium; jitse.creemers@uantwerpen.be (J.C.); marcel.eens@uantwerpen.be (M.E.); ruben.evens@uclouvain.be (R.E.)

2 Department of Zoology, Faculty of Natural Sciences, Comenius University, Ilkovičova 6, 842 15 Bratislava, Slovakia

3 Research Group: Zoology, Biodiversity and Toxicology, Hasselt University, Centre for Environmental Sciences, Campus Diepenbeek, Agoralaan, Gebouw D, 3590 Diepenbeek, Belgium; rhune.vandeneynde@student.uhasselt.be (R.V.E.); michiel.lathouwers@uhasselt.be (M.L.)

4 Terrestrial Ecology and Biodiversity Conservation Group, Earth and Life Institute, Université Catholique de Louvain, Croix du Sud 4-5, 1348 Louvain-la-Neuve, Belgium

5 Agentschap Natuur en Bos, Regio Noord-Limburg, Heuvelstraat 1C, 3941 Hechel-Eksel, Belgium; eddy.ulenaers@vlaanderen.be

6 Department of Geography, Institute of Life, Earth and Environment (ILEE), University of Namur, 61 Rue de Bruxelles, 5000 Namur, Belgium

* Correspondence: richard.schnurmacher@uantwerpen.be

† These authors contributed equally to this work.

‡ These authors contributed equally to this work.

Supplementary Materials

Figures

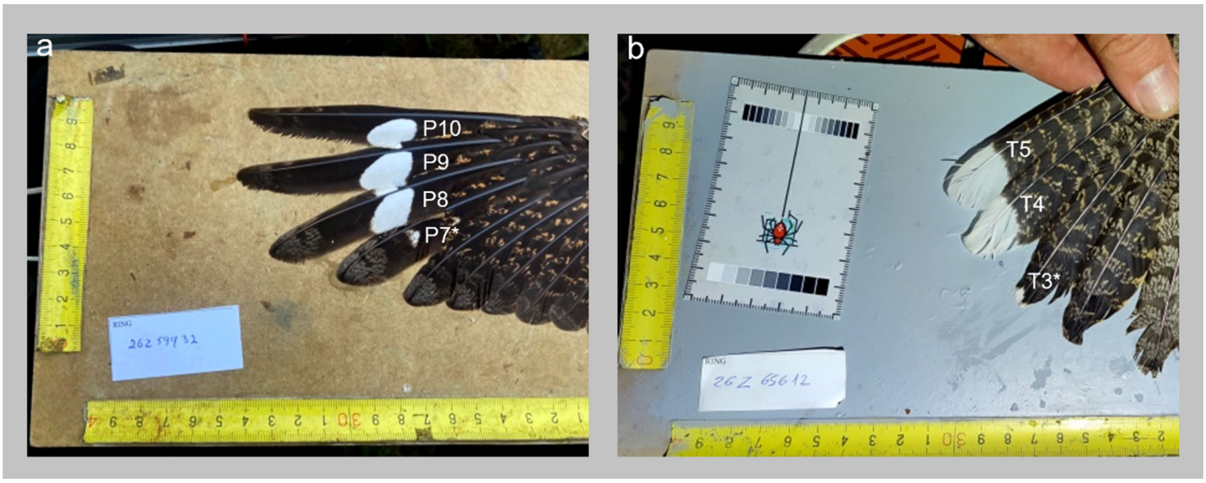

Figure S1. *White markings on the outer (**a**) primaries (P8–P10) and (**b**) rectrices (T4, T5) of a male Nightjar. Small additional markings on the feathers denoted with asterisk (P7* and T3*) are exceptionally rare, occurring in fewer than 1% of all captured males (6 out of 736) and were never recorded simultaneously.*

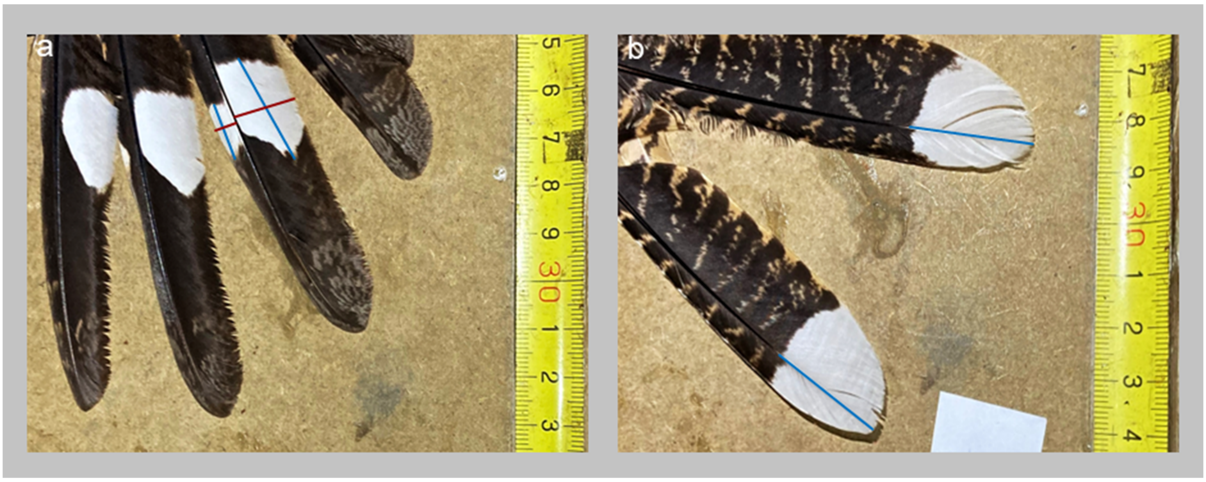

Figure S2. *Manual measurements of feather markings: (**a**) measurement of the surface area of the markings in primary P8. The blue lines represent the measured heights of the inner and outer webs, whereas the red lines show the widths. The surface area was calculated as the sum of height multiplied by the width of each web; (**b**) measurement of the marking height in the rectrices T4 and T5, with the heights represented by blue lines.*

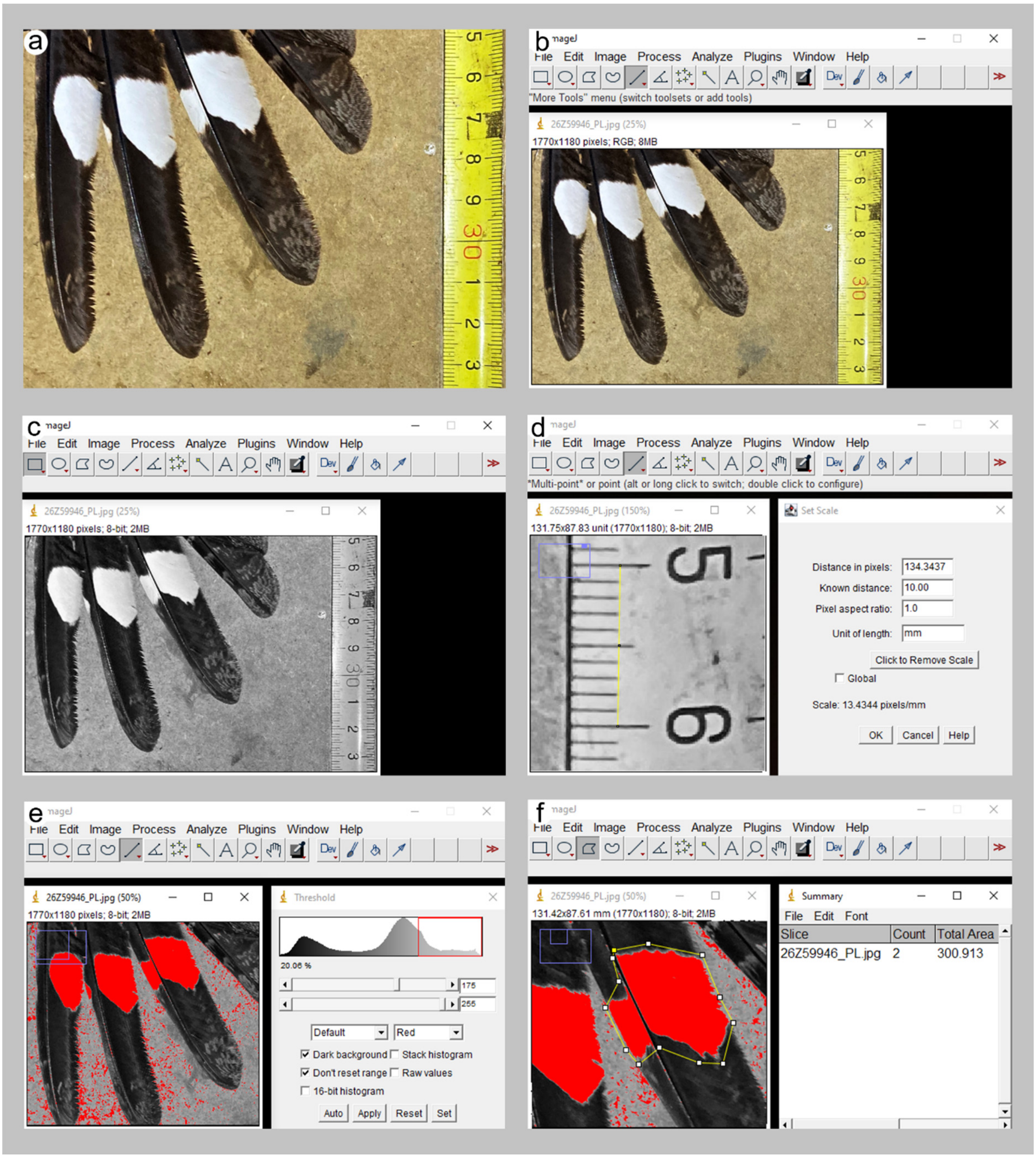

Figure S3. *The protocol followed to obtain digital measurements of the feather markings: (**a**) clear original photo of the three outermost left primaries prepared for import to ImageJ; (**b**) photo imported into ImageJ by dragging the file to the ImageJ window; (**c**) image type converted from RGB to 8-bit for further analysis (Image → Type → 8-bit); (**d**) zooming in and dragging a straight line to set the scale for 10 mm from the tape measure, then changing the known distance to '10' and the unit of length to 'mm' (analyse → set scale…); (**e**) adjusting the threshold using the upper sliding feature for particle analysis, so the white area of the markings is marked red (image → adjust → threshold); (**f**) marking the surface area of the respective markings (here P8) with the polygon tool and analysing the particles to obtain the total area of the selected marking in mm^2 (Analyse → Analyse particles).*

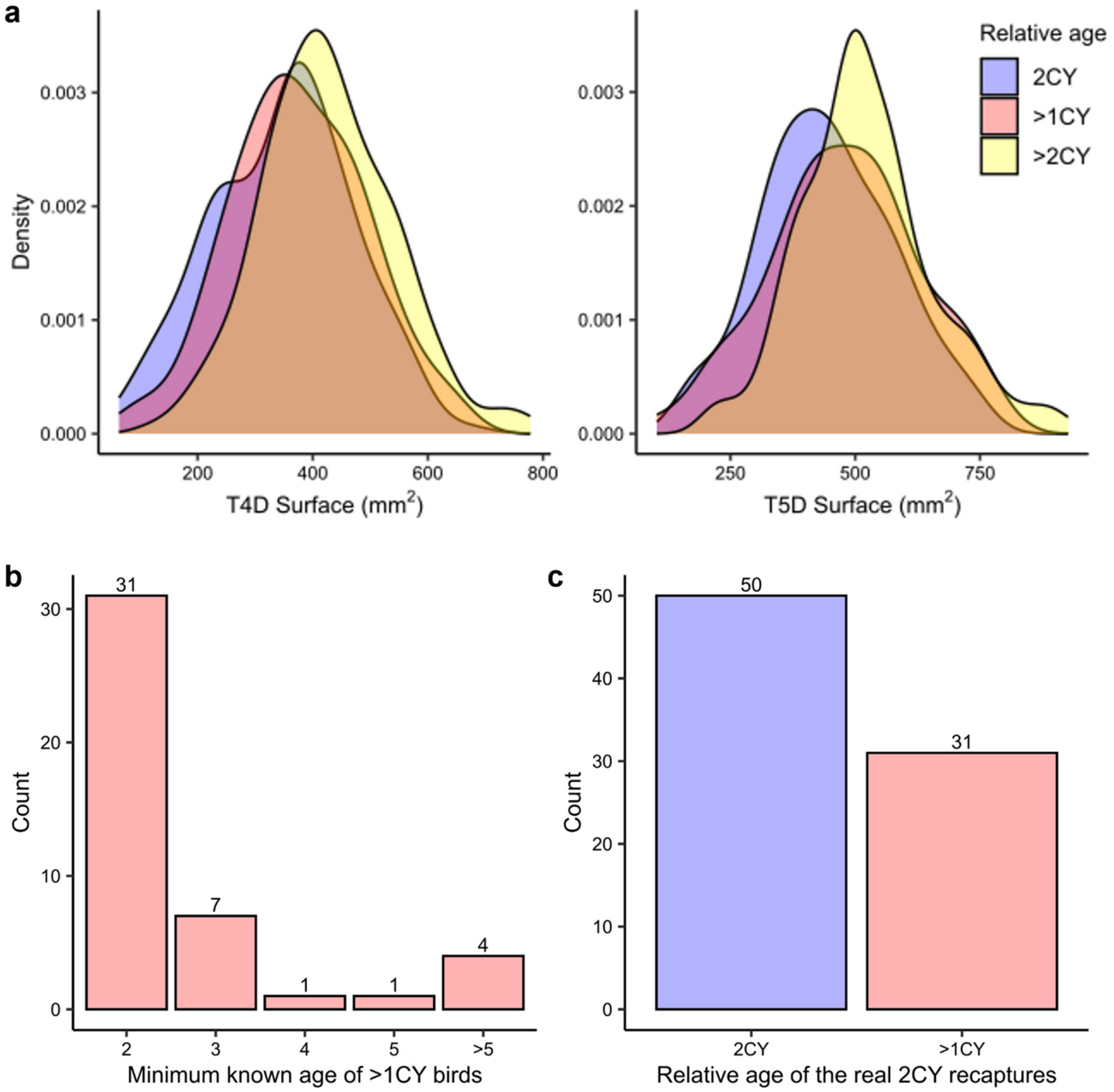

Figure S4. *(**a**) Density plots of the tail feather surface of male Nightjars by age group, suggesting that >1CY individuals comprise both 2CY and >2CY birds, with more individuals originating from 2CY cohort. This is supported by the categorisation of these individuals in data on recaptured individuals. (**b**) Most recaptured individuals aged as >1CY were, in fact, 2CYs based on their age during the initial ringing date. (**c**) On the contrary, >1CY birds form a significant proportion of actual yearling birds from the recapture data.*

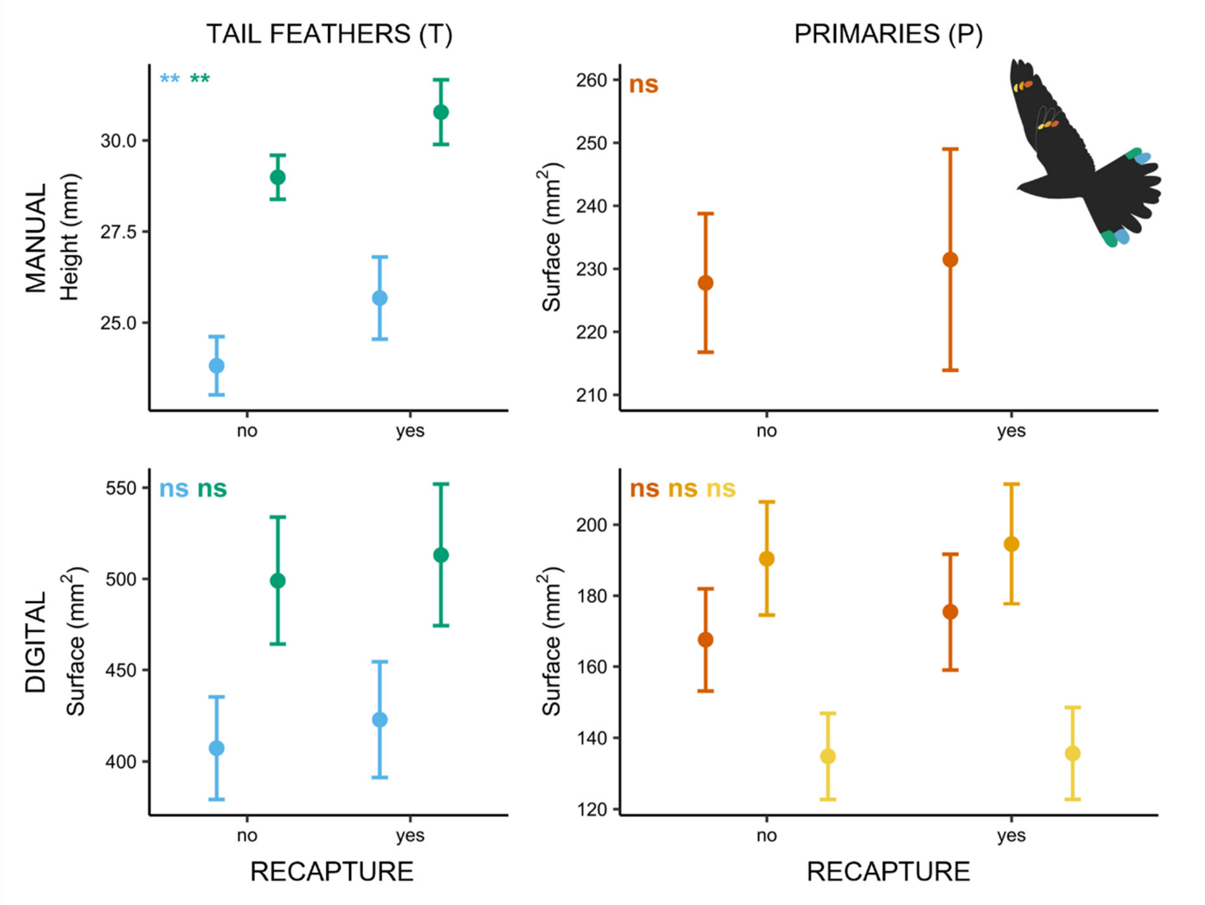

Figure S5. *Marking size of male Nightjars in relation to their site fidelity. Notice the significant difference in height of the tail markings; recaptured males generally have larger markings. The inscriptions in the upper left corner indicate the overall significance of the models with matching colours. The colours correspond to the respective feather markings in the flying Nightjar icon.*

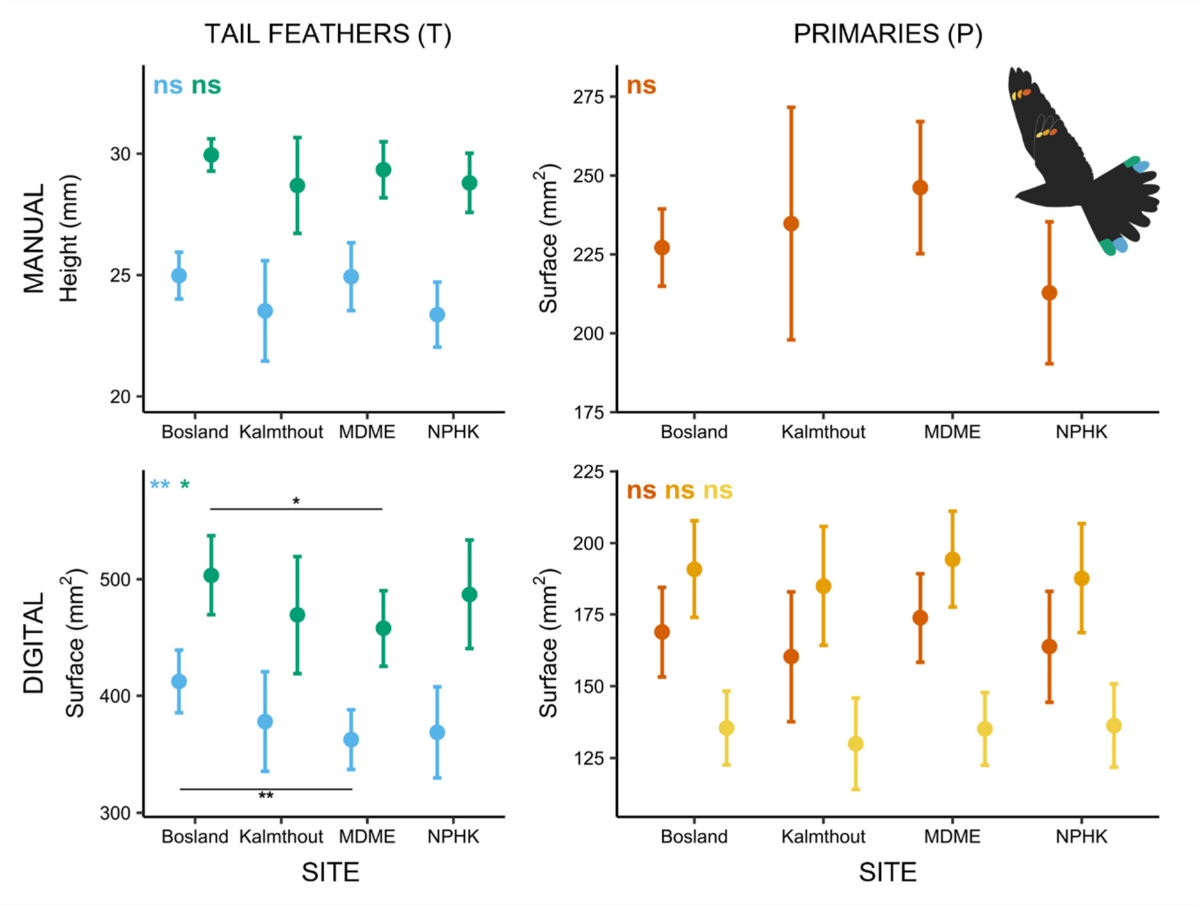

Figure S6. *Marking size differences of male Nightjars between study sites. The models were significant for the surface areas of both rectrices, with post hoc tests indicating marked differences between Bosland and MDME. Inscriptions in the upper left corner indicate the overall significance of the models with matching colours, horizontal lines with asterisks mark significant post hoc results. The colours correspond to the respective feather markings in the flying Nightjar icon.*

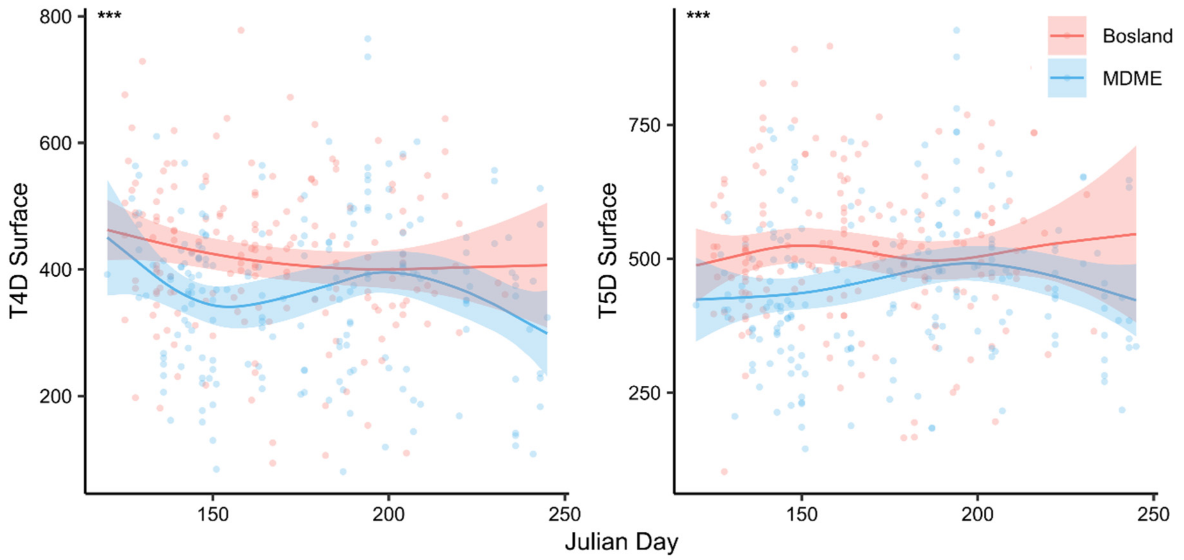

Figure S7. *Comparison of changes in within-year marking size between Bosland and MDME by date of capture, showing greater fluctuation in MDME. The asterisks in the upper left corner indicate the overall significance of the population marking size differences between Bosland (red) and MDME (blue), with raw data points represented as full circles. The Julian Day range represents the span of the recorded individuals, with the ranges of the respective months, as follows: 115–120 (beginning 25 April); 121–151 (May); 152–181 (June); 182–212 (July); 213–243 (August); and 244–250 (ending 7 September).*

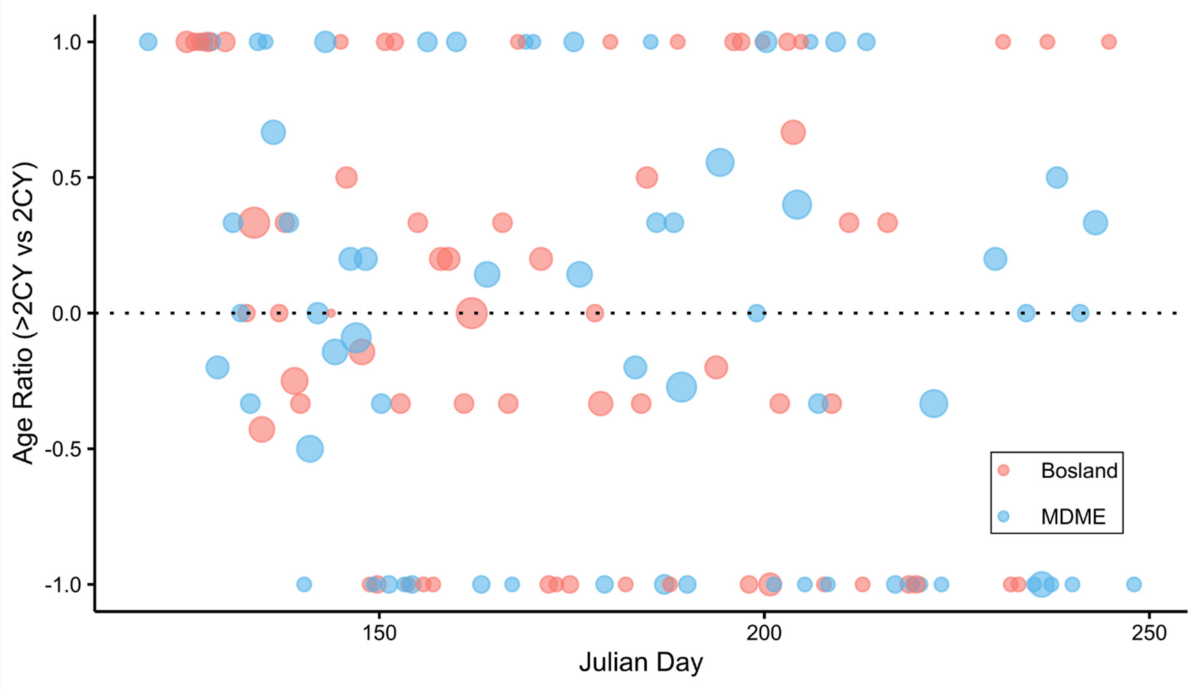

Figure S8. *Ratios of >2CY (positive values) and 2CY (negative values) of male Nightjars caught throughout the season in Bosland (red circles) and MDME (blue circles). The circle sizes represent the total number of birds caught per Julian Day (1 to 12 individuals). Note the prevalence of 2CY males in late May and at the end of the season, especially in MDME. The Julian Day range represents the span of the recorded individuals, with the ranges of the respective months, as follows: 115–120 (beginning 25 April); 121–151 (May); 152–181 (June); 182–212 (July); 213–243 (August); and 244–250 (ending 7 September).*

Tables

Table S1. *Overview of the total number of male European Nightjars captured each year at the respective study sites by age group. Note that some individuals were caught repeatedly between years (N = 955 between-year captures of 736 unique birds). Study sites: Bosland–National Park Bosland; Kalmthout—Grenspark Kalmthoutse Heide; MDME—Oudsbergen Military Area; NPHK—National Park Hoge Kempen.*

Manual

Year	Site	Age 2CY	Age >1CY	Age >2CY	Total
2010		**8**	**6**	**0**	**14**
	Bosland	8	6	0	14
2011		**7**	**13**	**5**	**25**
	Bosland	7	9	3	19
	MDME	0	4	2	6
2012		**7**	**3**	**8**	**18**
	Bosland	6	3	6	15
	MDME	1	0	2	3
2013		**4**	**2**	**16**	**22**
	Bosland	4	2	15	21
	MDME	0	0	1	1
2014		**12**	**3**	**25**	**40**
	Bosland	7	1	20	28
	MDME	5	2	2	9
	NPHK	0	0	3	3
2015		**15**	**6**	**32**	**53**
	Bosland	10	3	16	29
	MDME	3	0	8	11
	NPHK	2	3	8	13
2016		**23**	**13**	**45**	**81**
	Bosland	14	5	23	42
	MDME	4	4	9	17
	NPHK	5	4	13	22
2017		**19**	**9**	**41**	**69**
	Bosland	7	5	15	27
	Kalmthout	3	0	2	5
	MDME	6	3	11	20
	NPHK	3	1	13	17
2018		**12**	**0**	**9**	**21**
	Bosland	2	0	4	6
	Kalmthout	9	0	5	14
	MDME	1	0	0	1
Total		**107**	**55**	**181**	**343**

Digital

Year	Site	Age 2CY	Age >1CY	Age >2CY	Total
2019		**4**	**1**	**3**	**8**
	Bosland	1	1	1	3
	MDME	3	0	2	5
2020		**63**	**22**	**76**	**161**
	Bosland	34	9	33	76
	Kalmthout	1	2	1	4
	MDME	16	4	21	41
	NPHK	12	7	21	40
2021		**60**	**21**	**52**	**133**
	Bosland	24	10	17	51
	Kalmthout	2	2	0	4
	MDME	28	5	14	47
	NPHK	6	4	21	31
2022		**22**	**12**	**42**	**76**
	Bosland	9	6	16	31
	Kalmthout	0	1	7	8
	MDME	11	3	17	31
	NPHK	2	2	2	6
2023		**37**	**11**	**38**	**86**
	Bosland	9	4	13	26
	Kalmthout	8	2	7	17
	MDME	20	5	15	40
	NPHK	0	0	3	3
2024		**47**	**23**	**78**	**148**
	Bosland	13	9	19	41
	Kalmthout	7	4	9	20
	MDME	27	10	47	84
	NPHK	0	0	3	3
Total		**233**	**90**	**289**	**612**

Table S2. *Overview of the total number of male European Nightjars recaptured across years at the respective study sites by age group. Note that some individuals were recaptured more than once. Study sites: Bosland—National Park Bosland; MDME—Oudsbergen Military Area; NPHK—National Park Hoge Kempen. Site Kalmthout—Grenspark Kalmthoutse Heide was omitted from the recapture dataset analysis due to the insufficient sample size (N = 11).*

Manual

Year	Site	Age 2CY	>1CY	>2CY	Total
2010		**3**	**3**	**0**	**6**
	Bosland	3	3	0	6
2011		**2**	**6**	**1**	**9**
	Bosland	2	6	1	9
2012		**0**	**0**	**6**	**6**
	Bosland	0	0	4	4
	MDME	0	0	2	2
2013		**1**	**0**	**7**	**8**
	Bosland	1	0	7	8
2014		**4**	**1**	**15**	**20**
	Bosland	2	1	12	15
	MDME	2	0	2	4
	NPHK	0	0	1	1
2015		**3**	**2**	**20**	**25**
	Bosland	2	2	12	16
	MDME	1	0	3	4
	NPHK	0	0	5	5
2016		**4**	**4**	**29**	**37**
	Bosland	1	1	17	19
	MDME	2	2	6	10
	NPHK	1	1	6	8
2017		**2**	**3**	**26**	**31**
	Bosland	0	3	12	15
	MDME	1	0	8	9
	NPHK	1	0	6	7
2018		**1**	**1**	**0**	**2**
	Bosland	0	0	0	0
	MDME	1	1	0	2
Total		**20**	**20**	**104**	**144**

Digital

Year	Site	Age 2CY	>1CY	>2CY	Total
2019		**0**	**1**	**0**	**1**
	Bosland	0	1	0	1
2020		**13**	**7**	**34**	**54**
	Bosland	6	4	19	29
	MDME	5	1	8	14
	NPHK	2	2	7	11
		**8**	**7**	**37**	**52**
2021	*Bosland*	1	4	16	21
	MDME	7	2	8	17
	NPHK	0	1	13	14
2022		**5**	**4**	**26**	**35**
	Bosland	1	4	12	17
	MDME	3	0	12	15
	NPHK	1	0	2	3
2023		**0**	**0**	**18**	**18**
	Bosland	0	0	9	9
	MDME	0	0	6	6
	NPHK	0	0	3	3
2024		**2**	**4**	**33**	**39**
	Bosland	0	0	11	11
	MDME	2	4	22	28
Total		**28**	**23**	**148**	**199**

Table S3. *Results of compared values obtained from museum male Nightjar specimens (N = 22). The t-test comparison of left–right side marking symmetry of the P-values using t-tests shows a high degree of symmetry, all results are non-significant (P > 0.05). Linear regression of the single-side (left) data revealed strong correlations between the marking proportions measured manually and digitally measured marking proportions in the respective feathers, producing statistically significant results (P < 0.05).*

**Feather**	***T-test***		***Linear model***	
	Method		*Digital ~ Manual*	
	**Manual**	**Digital**	**Adj. R^2**	**P**
**T4**	0.90	0.60	0.291	0.0101
**T5**	0.43	0.62	0.840	<0.001
**P8**	0.95	0.93	0.878	<0.001
**P9**	0.48	0.43	0.725	<0.001
**P10**	0.78	0.88	0.662	<0.001

Table S4. *Pairwise comparisons of modified signed-likelihood ratio tests (MSLRTs) for coefficient of variation. Non-significant p-values are italicised, while significant p-values (< 0.05) indicate marked differences, particularly between marking sizes and morphometric traits. Note that the p-values of pairwise comparisons between the marking size and other morphometric traits were lower than those comparing two marking sizes or two other morphometric traits. Legend: T4M—manually measured T4; T4D—digitally measured T4; T5M—manually measured T5; T5D—digitally measured T4; P8M—manually measured P8; P8D—digitally measured P8; P9D—digitally measured P9; P10D—digitally measured P10; W—weight; SL—sternum length; TL1—length of the central tail feather T1; TL5—length of the outer tail feather T5; WL—wing length.*

Traits	T4M	T4D	T5M	T5D	P8M	P8D	P9D	P10D	W	SL	TL1	TL5	WL
**T4M**	1	-	-	-	-	-	-	-	-	-	-	-	-
**T4D**	<0.001	1	-	-	-	-	-	-	-	-	-	-	-
**T5M**	<0.001	<0.001	1	-	-	-	-	-	-	-	-	-	-
**T5D**	<0.001	0.049	<0.001	1	-	-	-	-	-	-	-	-	-
**P8M**	<0.001	*0.268*	<0.001	0.004	1	-	-	-	-	-	-	-	-
**P8D**	<0.001	*0.109*	<0.001	<0.001	*0.772*	1	-	-	-	-	-	-	-
**P9D**	<0.001	<0.001	<0.001	<0.001	<0.001	<0.001	1	-	-	-	-	-	-
**P10D**	<0.001	<0.001	<0.001	0.016	<0.001	<0.001	0.037	1	-	-	-	-	-
**W**	<0.001	<0.001	<0.001	<0.001	<0.001	<0.001	<0.001	<0.001	1	-	-	-	-
**SL**	<0.001	<0.001	<0.001	<0.001	<0.001	<0.001	<0.001	<0.001	*0.583*	1	-	-	-
**TL1**	<0.001	<0.001	<0.001	<0.001	<0.001	<0.001	<0.001	<0.001	<0.001	<0.001	1	-	-
**TL5**	<0.001	<0.001	<0.001	<0.001	<0.001	<0.001	<0.001	<0.001	<0.001	<0.001	<0.001	1	-
**WL**	<0.001	<0.001	<0.001	<0.001	<0.001	<0.001	<0.001	<0.001	<0.001	<0.001	<0.001	<0.001	1

Table S5. *Results of generalised linear mixed models, type III analyses of variance and significant post hoc tests, showing effects of relative age on size of the white tail markings of male Nightjars.*

	Model 1. Manual length T4 marking							
	Conditional model					*Anova*		
**Predictors**	**Estimate**	**SE**		**z**	**P**	**χ^2**	**df**	**P**
Intercept	24.366	0.529		46.020	<0.001	2118.164	1	<0.001
Relative age						1.329	2	0.515
>1CY	-0.636	0.936		-0.680	0.497			
>2CY	0.293	0.632		0.460	0.643			
**Random effect**	**Variance**	**SD**						
Individual ID	15.739	3.967						
	Model 2. Manual length T5 marking							
	Conditional model					*Anova*		
**Predictors**	**Estimate**	**SE**		**z**	**P**	**χ^2**	**df**	**P**
Intercept	28.753	0.403		71.410	<0.001	5099.550	1	<0.001
Relative age						10.030	2	0.007
>1CY	0.430	0.655		0.660	0.511			
>2CY	1.479	0.493		3.000	0.003			
**Random effect**	**Variance**	**SD**						
Individual ID	11.211	3.348						
	Significant post hoc tests							
**Contrast**	**Estimate**	**SE**	**df**	**t-ratio**	**P**			
Relative age								
2CY *vs* >2CY	-1.479	0.493	328	-2.999	0.009			
	Model 3. Digital surface area T4 marking							
	Conditional model					*Anova*		
**Predictors**	**Estimate**	**SE**		**z**	**P**	**χ^2**	**df**	**P**
Intercept	378.320	13.970		27.078	<0.001	733.206	1	<0.001
Relative age						54.920	2	<0.001
>1CY	20.920	15.400		1.358	0.174			
>2CY	78.120	10.890		7.175	<0.001			
Site								
Kalmthout	-33.450	21.060		-1.588	0.112	18.044	3	<0.001
MDME	-50.710	13.160		-3.854	<0.001			
NPHK	-56.790	19.370		-2.931	0.003			
**Random effect**	**Variance**	**SD**						
Individual ID	7702.700	87.770						
Year	332.600	18.240						
	Significant post hoc tests							
**Contrast**	**Estimate**	**SE**	**df**	**t-ratio**	**P**			
Relative age								
2CY *vs* >2CY	-78.118	10.887	460	-7.175	<0.001			
>1CY *vs* >2CY	-57.201	14.621	460	-3.912	<0.001			
Site								
Bosland *vs* MDME	50.712	13.157	460	3.854	<0.001			
Bosland *vs* NPHK	56.789	19.375	460	2.931	0.021			
	Model 4. Digital surface area T5 marking							
	Conditional model					*Anova*		
**Predictors**	**Estimate**	**SE**		**z**	**P**	**χ^2**	**df**	**P**
Intercept	472.080	18.420		25.633	<0.001	657.031	1	<0.001
Relative age						32.589	2	<0.001
>1CY	23.270	17.610		1.322	0.186			
>2CY	69.240	12.390		5.589	<0.001			
Site						9.400	3	0.024
Kalmthout	-32.620	23.920		-1.364	0.173			
MDME	-45.210	14.960		-3.021	0.003			
NPHK	-29.430	21.960		-1.340	0.180			
**Random effect**	**Variance**	**SD**						
Individual ID	9879.100	99.390						
Year	912.100	30.200						
	Significant post hoc tests							
**Contrast**	**Estimate**	**SE**	**df**	**t-ratio**	**P**			
Relative age								
2CY *vs* >2CY	-69.245	12.389	462	-5.589	<0.001			
>1CY *vs* >2CY	-45.970	16.681	462	-2.756	0.018			
Site								
Bosland *vs* MDME	45.214	14.965	462	3.021	0.016			

Table S6. *Results of generalised linear mixed models, type III analyses of variance and significant post hoc tests, showing effects of relative age on size of the white wing markings of male Nightjars.*

Model 5. Manual surface area P8 marking

	Conditional model				*Anova*		
**Predictors**	**Estimate**	**SE**	**z**	**P**	**χ²**	**df**	**P**
Intercept	221.656	6.498	34.110	<0.001	1163.437	1	<0.001
Relative age					2.653	2	0.265
>1CY	13.375	9.370	1.430	0.153			
>2CY	10.513	7.208	1.460	0.145			
**Random effect**	**Variance**	**SD**					
Individual ID	5108.400	71.470					

Model 6. Digital surface area P8 marking

	Conditional model				*Anova*		
**Predictors**	**Estimate**	**SE**	**z**	**P**	**χ²**	**df**	**P**
Intercept	165.551	7.081	23.379	<0.001	546.568	1	<0.001
Relative age					4.421	2	0.110
>1CY	4.717	6.179	0.763	0.445			
>2CY	8.937	4.269	2.093	0.036			
**Random effect**	**Variance**	**SD**					
Individual ID	2719.000	52.140					
Year	196.700	14.030					

Model 7. Digital surface area P9 marking

	Conditional model				*Anova*		
**Predictors**	**Estimate**	**SE**	**z**	**P**	**χ²**	**df**	**P**
Intercept	186.157	7.989	23.301	<0.001	542.935	1	<0.001
Relative age					9.731	2	0.008
>1CY	5.629	5.206	1.081	0.280			
>2CY	11.303	3.635	3.109	0.002			
**Random effect**	**Variance**	**SD**					
Individual ID	1121.900	33.500					
Year	304.700	17.460					

Significant post hoc tests

**Contrast**	**Estimate**	**SE**	**df**	**t-ratio**	**P**
Relative age					
2CY *vs* >2CY	-11.303	3.635	604	-3.109	0.006

Model 8. Digital surface area P10 marking

	Conditional model				*Anova*		
**Predictors**	**Estimate**	**SE**	**z**	**P**	**χ²**	**df**	**P**
Intercept	132.703	6.132	21.641	<0.001	468.352	1	<0.001
Relative age					3.579	2	0.167
>1CY	1.531	4.009	0.382	0.703			
>2CY	5.084	2.763	1.840	0.066			
**Random effect**	**Variance**	**SD**					
Individual ID	756.200	27.500					
Year	174.100	13.200					

Table S7. *Results of generalised linear mixed models, type III analyses of variance and significant post hoc tests, showing effects of minimum known age on size of the white tail markings of male Nightjars.*

Model 1. Manual length T4 marking								
	Conditional model					*Anova*		
**Predictors**	**Estimate**	**SE**		**z**	**P**	**χ²**	**df**	**P**
Intercept	24.760	0.852		29.062	<0.001	844.590	1	<0.001
Minimum age						6.036	4	0.197
3CY	0.268	0.844		0.317	0.751			
4CY	0.774	0.948		0.817	0.414			
5CY	1.495	1.254		1.192	0.233			
>5CY	3.178	1.481		2.146	0.032			
**Random effect**	**Variance**	**SD**						
Individual ID	11.847	3.442						
Model 2. Manual length T5 marking								
	Conditional model					*Anova*		
**Predictors**	**Estimate**	**SE**		**z**	**P**	**χ²**	**df**	**P**
Intercept	29.785	0.664		44.850	<0.001	2011.349	1	<0.001
Minimum age						9.300	4	0.054
3CY	0.668	0.683		0.980	0.328			
4CY	1.711	0.817		2.100	0.036			
5CY	2.263	0.973		2.330	0.020			
>5CY	1.916	0.987		1.940	0.052			
**Random effect**	**Variance**	**SD**						
Individual ID	11.742	3.427						
Model 3. Digital surface area T4 marking								
	Conditional model					*Anova*		
**Predictors**	**Estimate**	**SE**		**z**	**P**	**χ²**	**df**	**P**
Intercept	355.310	28.210		12.595	<0.001	158.631	1	<0.001
Minimum age						29.743	4	<0.001
3CY	79.640	18.820		4.231	<0.001			
4CY	118.390	22.870		5.176	<0.001			
5CY	85.600	29.300		2.921	0.003			
>5CY	78.800	30.400		2.592	0.010			
Site						8.361	2	0.015
MDME	-60.250	22.640		-2.661	0.008			
NPHK	-60.790	31.070		-1.956	0.050			
**Random effect**	**Variance**	**SD**						
Individual ID	6247.000	79.040						
Year	1330.000	36.460						
	Significant post hoc tests							
**Contrast**	**Estimate**	**SE**	**df**	**t-ratio**	**P**			
Minimum age								
2CY *vs* 3CY	-79.639	18.824	142	-4.231	<0.001			
2CY *vs* 4CY	-118.385	22.872	142	-5.176	<0.001			
2CY *vs* 5CY	-85.598	29.301	142	-2.921	0.040			
Site								
Bosland *vs* MDME	60.247	22.643	142	2.661	0.026			
Model 4. Digital surface area T5 marking								
	Conditional model					*Anova*		
**Predictors**	**Estimate**	**SE**		**z**	**P**	**χ²**	**df**	**P**
Intercept	487.980	31.600		15.445	<0.001	238.536	1	<0.001
Minimum age						9.956	4	0.041
3CY	56.340	22.160		2.543	0.011			
4CY	68.480	27.110		2.526	0.012			
5CY	31.830	35.200		0.904	0.366			
>5CY	79.010	37.050		2.133	0.033			
Site						11.149	2	0.004
MDME	-93.100	28.410		-3.277	0.001			
NPHK	-20.770	39.080		-0.531	0.595			
**Random effect**	**Variance**	**SD**						
Individual ID	10732.000	103.590						
Year	1162.000	34.080						
	Significant post hoc tests							
**Contrast**	**Estimate**	**SE**	**df**	**t-ratio**	**P**			
Site								
Bosland *vs* MDME	93.095	28.406	143	3.277	0.004			

Table S8. *Results of generalised linear mixed models, type III analyses of variance and significant post hoc tests, showing effects of minimum known age on size of the white tail markings of male Nightjars.*

		Model 5. Manual surface area P8 marking						
	Conditional model					*Anova*		
**Predictors**	**Estimate**	**SE**		**z**	**P**	**χ^2**	**df**	**P**
Intercept	231.688	10.492		22.082	<0.001	0.595	4	0.964
Minimum age								
3CY	-3.404	7.806		-0.436	0.663			
4CY	-1.842	9.468		-0.195	0.846			
5CY	-5.872	10.940		-0.537	0.591			
>5CY	2.710	11.549		0.235	0.814			
**Random effect**	**Variance**	**SD**						
Individual ID	5252.800	72.480						
		Model 6. Digital surface area P8 marking						
	Conditional model					*Anova*		
**Predictors**	**Estimate**	**SE**		**z**	**P**	**χ^2**	**df**	**P**
Intercept	167.201	9.330		17.921	<0.001	321.155	1	<0.001
Minimum age						6.127	4	0.190
3CY	8.745	5.359		1.632	0.103			
4CY	13.814	7.783		1.775	0.076			
5CY	9.826	11.598		0.847	0.397			
>5CY	29.019	13.116		2.213	0.027			
**Random effect**	**Variance**	**SD**						
Individual ID	3750.400	61.240						
Year	117.000	10.820						
		Model 7. Digital surface area P9 marking						
	Conditional model					*Anova*		
**Predictors**	**Estimate**	**SE**		**z**	**P**	**χ^2**	**df**	**P**
Intercept	191.270	10.898		17.550	<0.001	308.019	1	<0.001
Minimum age						5.412	4	0.248
3CY	8.192	5.664		1.446	0.148			
4CY	17.440	7.917		2.203	0.028			
5CY	6.654	11.506		0.578	0.563			
>5CY	9.938	12.007		0.828	0.408			
**Random effect**	**Variance**	**SD**						
Individual ID	2616.500	51.150						
Year	339.400	18.420						
		Model 8. Digital surface area P10 marking						
	Conditional model					*Anova*		
**Predictors**	**Estimate**	**SE**		**z**	**P**	**χ^2**	**df**	**P**
Intercept	128.853	6.951		18.538	<0.001	343.658	1	<0.001
Minimum age						9.173	4	0.057
3CY	6.592	4.667		1.412	0.158			
4CY	11.227	6.167		1.820	0.069			
5CY	10.519	8.537		1.232	0.218			
>5CY	23.786	8.110		2.933	0.003			
**Random effect**	**Variance**	**SD**						
Individual ID	827.800	28.770						
Year	107.000	10.350						
	Significant post hoc tests							
**Contrast**	**Estimate**	**SE**	**df**	**t-ratio**	**P**			
Minimum age								
2CY *vs* >5CY	-23.786	8.110	189	-2.933	0.037			

Table S9. *Results of generalised linear mixed models, type III analyses of variance and significant post hoc tests, showing effects of body condition index on size of the white tail markings of male Nightjars.*

Model 1. Manual length T4 marking								
	Conditional model					*Anova*		
**Predictors**	**Estimate**	**SE**		**z**	**P**	**χ^2**	**df**	**P**
Intercept	24.620	0.348		70.720	<0.001	5001.082	1	<0.001
Body condition	0.173	0.073		2.380	0.017	5.658	1	0.017
**Random effect**	**Variance**	**SD**						
Individual ID	15.378	3.921						
Model 2. Manual length T5 marking								
	Conditional model					*Anova*		
**Predictors**	**Estimate**	**SE**		**z**	**P**	**χ^2**	**df**	**P**
Intercept	29.764	0.264		112.570	<0.001	12670.886	1	<0.001
Body condition	0.208	0.056		3.690	<0.001	13.617	1	<0.001
**Random effect**	**Variance**	**SD**						
Individual ID	11.760	3.429						
Model 3. Digital surface area T4 marking								
	Conditional model					*Anova*		
**Predictors**	**Estimate**	**SE**		**z**	**P**	**χ^2**	**df**	**P**
Intercept	410.845	14.684		27.979	<0.001	782.807	1	<0.001
Body condition	4.047	1.257		3.220	0.001	10.367	1	0.001
Site						13.211	3	0.004
Kalmthout	-35.489	21.825		-1.626	0.104			
MDME	-45.446	13.693		-3.319	<0.001			
NPHK	-48.880	20.554		-2.378	0.017			
**Random effect**	**Variance**	**SD**						
Individual ID	7829.400	88.480						
Year	589.300	24.280						
	Significant post hoc tests							
**Contrast**	**Estimate**	**SE**	**df**	**t-ratio**	**P**			
Site								
Bosland *vs* MDME	45.446	13.693	449	3.319	0.006			
Model 4. Digital surface area T5 marking								
	Conditional model					*Anova*		
**Predictors**	**Estimate**	**SE**		**z**	**P**	**χ^2**	**df**	**P**
Intercept	477.320	17.070		27.962	<0.001	781.892	1	<0.001
Body condition	3.989	1.389		2.871	0.004	8.241	1	0.004
**Random effect**	**Variance**	**SD**						
Individual ID	10809	103.970						
Year	1310	36.190						

Table S10. *Results of generalised linear mixed models and type III analyses of variance, showing effects of body condition index on size of the white wing markings of male Nightjars.*

Model 5. Manual surface area P8 marking							
	Conditional model				*Anova*		
**Predictors**	**Estimate**	**SE**	**z**	**P**	**χ^2**	**df**	**P**
Intercept	230.444	4.786	48.150	<0.001	2318.753	1	<0.001
Body condition	1.776	0.968	1.830	0.067	3.365	1	0.067
**Random effect**	**Variance**	**SD**					
Individual ID	4913.900	70.100					
Model 6. Digital surface area P8 marking							
	Conditional model				*Anova*		
**Predictors**	**Estimate**	**SE**	**z**	**P**	**χ^2**	**df**	**P**
Intercept	169.363	7.392	22.912	<0.001	524.974	1	<0.001
Body condition	1.719	0.512	3.359	<0.001	11.285	1	<0.001
**Random effect**	**Variance**	**SD**					
Individual ID	2683.700	51.800					
Year	253.800	15.930					
Model 7. Digital surface area P9 marking							
	Conditional model				*Anova*		
**Predictors**	**Estimate**	**SE**	**z**	**P**	**χ^2**	**df**	**P**
Intercept	191.974	8.072	23.783	<0.001	565.626	1	<0.001
Body condition	1.689	0.396	4.263	<0.001	18.172	1	<0.001
**Random effect**	**Variance**	**SD**					
Individual ID	1089	33					
Year	338.100	18.390					
Model 8. Digital surface area P10 marking							
	Conditional model				*Anova*		
**Predictors**	**Estimate**	**SE**	**z**	**P**	**χ^2**	**df**	**P**
Intercept	135.285	6.168	21.933	<0.001	481.065	1	<0.001
Body condition	0.778	0.307	2.533	0.011	6.417	1	0.011
**Random effect**	**Variance**	**SD**					
Individual ID	748	27.350					
Year	190.700	13.810					

Table S11. *Results of generalised linear mixed models, type III analyses of variance and significant post hoc tests, showing effects of site fidelity (recapture: yes/no) on size of the white tail markings of male Nightjars.*

	Model 1. Manual length T4 marking							
	Conditional model					*Anova*		
**Predictors**	**Estimate**	**SE**		**z**	**P**	**χ^2**	**df**	**P**
Intercept	23.818	0.407		58.490	<0.001	3420.826	1	<0.001
Recapture						6.953	1	0.008
yes	1.858	0.705		2.640	0.008			
**Random effect**	**Variance**	**SD**						
Individual ID	15.030	3.877						
	Model 2. Manual length T5 marking							
	Conditional model					*Anova*		
**Predictors**	**Estimate**	**SE**		**z**	**P**	**χ^2**	**df**	**P**
Intercept	28.989	0.308		94.230	<0.001	8879.826	1	<0.001
Recapture						10.664	1	0.001
yes	1.787	0.547		3.270	0.001			
**Random effect**	**Variance**	**SD**						
Individual ID	10.471	3.236						
	Model 3. Digital surface area T4 marking							
	Conditional model					*Anova*		
**Predictors**	**Estimate**	**SE**		**z**	**P**	**χ^2**	**df**	**P**
Intercept	407.260	14.270		28.538	<0.001	814.422	1	<0.001
Recapture						1.540	1	0.215
yes	15.590	12.560		1.241	0.215			
Site						14.822	3	0.002
Kalmthout	-35.940	21.960		-1.637	0.102			
MDME	-50.560	13.670		-3.698	<0.001			
NPHK	-43.900	20.130		-2.181	0.029			
**Random effect**	**Variance**	**SD**						
Individual ID	7602.700	87.190						
Year	434.300	20.840						
	Significant post hoc tests							
**Contrast**	**Estimate**	**SE**	**df**	**t-ratio**	**P**			
Site								
Bosland *vs* MDME	50.557	13.672	461	3.698	0.001			
	Model 4. Digital surface area T5 marking							
	Conditional model					*Anova*		
**Predictors**	**Estimate**	**SE**		**z**	**P**	**χ^2**	**df**	**P**
Intercept	498.990	17.790		28.044	<0.001	786.452	1	<0.001
Recapture						0.992	1	0.319
yes	14.080	14.140		0.996	0.319			
Site						9.336	3	0.025
Kalmthout	-35.610	24.640		-1.445	0.148			
MDME	-46.380	15.360		-3.019	0.003			
NPHK	-16.600	22.470		-0.739	0.460			
**Random effect**	**Variance**	**SD**						
Individual ID	10222.700	101.110						
Year	891.300	29.860						
	Significant post hoc tests							
**Contrast**	**Estimate**	**SE**	**df**	**t-ratio**	**P**			
Site								
Bosland *vs* MDME	46.383	15.363	463	3.019	0.016			

Table S12. *Results of generalised linear mixed models and type III analyses of variance, showing effects of site fidelity (recapture: yes/no) on size of the white wing markings of male Nightjars.*

Model 5. Manual surface area P8 marking							
	Conditional model				*Anova*		
**Predictors**	**Estimate**	**SE**	**z**	**P**	**χ^2**	**df**	**P**
Intercept	227.765	5.614	40.570	<0.001	1646.230	1	<0.001
Recapture					0.123	1	0.726
yes	3.702	10.568	0.350	0.726			
**Random effect**	**Variance**	**SD**					
Individual ID	5159.600	71.830					
Model 6. Digital surface area P8 marking							
	Conditional model				*Anova*		
**Predictors**	**Estimate**	**SE**	**z**	**P**	**χ^2**	**df**	**P**
Intercept	167.527	7.351	22.790	<0.001	519.366	1	<0.001
Recapture					1.810	1	0.179
yes	7.815	5.808	1.345	0.178			
**Random effect**	**Variance**	**SD**					
Individual ID	2711.700	52.070					
Year	233.500	15.280					
Model 7. Digital surface area P9 marking							
	Conditional model				*Anova*		
**Predictors**	**Estimate**	**SE**	**z**	**P**	**χ^2**	**df**	**P**
Intercept	190.409	8.160	23.334	<0.001	544.496	1	<0.001
Recapture					0.963	1	0.327
yes	4.146	4.226	0.981	0.326			
**Random effect**	**Variance**	**SD**					
Individual ID	1125.500	33.550					
Year	334.900	18.300					
Model 8. Digital surface area P10 marking							
	Conditional model				*Anova*		
**Predictors**	**Estimate**	**SE**	**z**	**P**	**χ^2**	**df**	**P**
Intercept	134.798	6.159	21.887	<0.001	479.047	1	<0.001
Recapture					0.062	1	0.804
yes	0.825	3.325	0.248	0.804			
**Random effect**	**Variance**	**SD**					
Individual ID	754.700	27.470					
Year	184.200	13.570					

Table S13. *Results of generalised linear mixed models, type III analyses of variance and significant post hoc tests, showing effects of the study site on size of the white tail markings of male Nightjars.*

			Model 1. Manual length T4 marking					
	Conditional model					*Anova*		
**Predictors**	**Estimate**	**SE**		**z**	**P**	**χ²**	**df**	**P**
Intercept	24.979	0.492		50.760	<0.001	2576.716	1	<0.001
Site						4.841	3	0.184
Kalmthout	-1.455	1.166		-1.250	0.212			
MDME	-0.048	0.854		-0.060	0.955			
NPHK	-1.611	0.842		-1.910	0.056			
**Random effect**	**Variance**	**SD**						
Individual ID	15.203	3.899						
			Model 2. Manual length T5 marking					
	Conditional model					*Anova*		
**Predictors**	**Estimate**	**SE**		**z**	**P**	**χ²**	**df**	**P**
Intercept	29.945	0.340		87.970	<0.001	7738.979	1	<0.001
Site						3.678	3	0.298
Kalmthout	-1.256	1.063		-1.180	0.237			
MDME	-0.606	0.676		-0.900	0.370			
NPHK	-1.148	0.710		-1.620	0.106			
**Random effect**	**Variance**	**SD**						
Individual ID	10.970	3.312						
			Model 3. Digital surface area T4 marking					
	Conditional model					*Anova*		
**Predictors**	**Estimate**	**SE**		**z**	**P**	**χ²**	**df**	**P**
Intercept	412.260	13.650		30.201	<0.001	912.107	1	<0.001
Site						14.323	3	0.002
Kalmthout	-34.330	21.980		-1.562	0.118			
MDME	-49.740	13.690		-3.633	<0.001			
NPHK	-43.570	20.180		-2.159	0.031			
**Random effect**	**Variance**	**SD**						
Individual ID	7696	87.730						
Year	423.900	20.590						
	Significant post hoc tests							
**Contrast**	**Estimate**	**SE**	**df**	**t-ratio**	**P**			
Site								
Bosland *vs* MDME	49.737	13.690	462	3.633	0.002			
			Model 4. Digital surface area T5 marking					
	Conditional model					*Anova*		
**Predictors**	**Estimate**	**SE**		**z**	**P**	**χ²**	**df**	**P**
Intercept	503.280	17.330		29.043	<0.001	843.520	1	<0.001
Site						8.981	3	0.030
Kalmthout	-34.130	24.640		-1.385	0.166			
MDME	-45.580	15.370		-2.965	0.003			
NPHK	-16.310	22.510		-0.725	0.469			
**Random effect**	**Variance**	**SD**						
Individual ID	10330.700	101.640						
Year	898.700	29.980						
	Significant post hoc tests							
**Contrast**	**Estimate**	**SE**	**df**	**t-ratio**	**P**			
Site								
Bosland *vs* MDME	45.577	15.371	464	2.965	0.019			

Table S14. *Results of generalised linear mixed models and type III analyses of variance, showing the effects of the study site on size of the white wing markings of male Nightjars.*

Model 5. Manual surface area P8 marking							
	Conditional model				*Anova*		
**Predictors**	**Estimate**	**SE**	**z**	**P**	**χ^2**	**df**	**P**
Intercept	227.145	6.245	36.370	<0.001	1322.984	1	<0.001
Site					4.778	3	0.189
Kalmthout	7.617	19.809	0.380	0.701			
MDME	19.027	12.255	1.550	0.121			
NPHK	-14.301	13.062	-1.090	0.274			
**Random effect**	**Variance**	**SD**					
Individual ID	5196.500	72.090					

Model 6. Digital surface area P8 marking							
	Conditional model				*Anova*		
**Predictors**	**Estimate**	**SE**	**z**	**P**	**χ^2**	**df**	**P**
Intercept	168.875	8.010	21.083	<0.001	444.485	1	<0.001
Site					2.809	3	0.422
Kalmthout	-8.562	10.270	-0.834	0.404			
MDME	4.962	5.939	0.835	0.403			
NPHK	-5.074	8.291	-0.612	0.541			
**Random effect**	**Variance**	**SD**					
Individual ID	2703.300	51.990					
Year	239	15.440					

Model 7. Digital surface area P9 marking							
	Conditional model				*Anova*		
**Predictors**	**Estimate**	**SE**	**z**	**P**	**χ^2**	**df**	**P**
Intercept	190.877	8.647	22.073	<0.001	487.227	1	<0.001
Site					2.323	3	0.508
Kalmthout	-5.866	7.643	-0.768	0.443			
MDME	3.454	4.463	0.774	0.439			
NPHK	-3.145	6.188	-0.508	0.611			
**Random effect**	**Variance**	**SD**					
Individual ID	1123.500	33.520					
Year	352.500	18.770					

Model 8. Digital surface area P10 marking							
	Conditional model				*Anova*		
**Predictors**	**Estimate**	**SE**	**z**	**P**	**χ^2**	**df**	**P**
Intercept	135.448	6.544	20.698	<0.001	428.408	1	<0.001
Site					0.983	3	0.805
Kalmthout	-5.491	5.968	-0.920	0.358			
MDME	-0.335	3.490	-0.096	0.924			
NPHK	0.845	4.828	0.175	0.861			
**Random effect**	**Variance**	**SD**					
Individual ID	756	27.500					
Year	193	13.890					
